# Sarcopenia Is Associated With a Risk of Mortality in People With Type 2 Diabetes Mellitus

**DOI:** 10.3389/fendo.2021.783363

**Published:** 2021-11-11

**Authors:** Fuyuko Takahashi, Yoshitaka Hashimoto, Ayumi Kaji, Ryosuke Sakai, Takuro Okamura, Noriyuki Kitagawa, Hiroshi Okada, Naoko Nakanishi, Saori Majima, Takafumi Senmaru, Emi Ushigome, Masahide Hamaguchi, Mai Asano, Masahiro Yamazaki, Michiaki Fukui

**Affiliations:** ^1^ Department of Endocrinology and Metabolism, Kyoto Prefectural University of Medicine, Graduate School of Medical Science, Kyoto, Japan; ^2^ Department of Diabetology, Kameoka Municipal Hospital, Kameoka, Japan; ^3^ Department of Diabetes and Endocrinology, Matsushita Memorial Hospital, Moriguchi, Japan

**Keywords:** sarcopenia, muscle mass, older, mortality, aged, diabetes

## Abstract

**Background:**

Sarcopenia has reportedly been associated with increased risk of mortality in general populations. However, few studies have investigated the association between sarcopenia and mortality in older people with type 2 diabetes mellitus (T2D). This study aimed to investigate the effect of sarcopenia on incident all-cause mortality in older people with T2D.

**Methods:**

Low muscle strength were set at handgrip strength <28 kg for men and <18 kg for women, and low skeletal muscle mass index (SMI), evaluated using the impedance body composition analyzer, were set at SMI <7.0 kg/m^2^ for men and <5.7 kg/m^2^ for women. People who had both low muscle strength and low SMI were diagnosed with sarcopenia. Due to a low incidence of all-cause mortality, the propensity score was used. The propensity score was evaluated using multivariable logistic regression models with the following parameters: age, sex, duration of diabetes, history of heart disease, history of cancer, smoking, exercise, alcohol, sodium-glucose cotransporter-2 inhibitor, glucagon-like peptide-1 receptor agonist, insulin, corticosteroid, hypertension, body mass index, glycosylated hemoglobin A1c, triglycerides, and creatinine, and the C-statistic was 0.89.

**Results:**

In this prospective cohort study, 396 people with an average age and duration of diabetes of 71.3 (6.3) years and 16.3 (11.3) years, respectively, were included. Of those included, 14.6% had sarcopenia. During the average 40.5 (16.5) months of follow-up, 13 people (6 out of the 338 without sarcopenia and 7 out of the 58 with sarcopenia) died. Incident rate were 5.1/1000 person years of follow-up in people without sarcopenia and 41.3/1000 person years of follow-up in people with sarcopenia. According to Cox regression analysis, sarcopenia was associated with all-cause mortality (adjusted hazard ratio: 6.12, 95% confidence interval: 1.52–24.7, *p* = 0.011).

**Conclusion:**

Sarcopenia is associated with incident all-cause mortality in older outpatients with T2D.

## Introdcution

The number of people with type 2 diabetes mellitus (T2D), including older people, has been on the increase worldwide (1). In older people, sarcopenia, which is defined as age-related loss of muscle mass, strength, and function (2), is of grave concern. According to the Asian Working Group for Sarcopenia (2), sarcopenia is characterized by muscle mass loss and low muscle strength or low physical performance. T2D has reportedly been associated with a 1.55-fold higher risk of sarcopenia in the older population than in the general population (3). Therefore, sarcopenia in people with T2D requires more attention than that in individuals without diabetes.

Sarcopenia has been associated with cardiometabolic diseases (CVD) (4–6), respiratory diseases (7), and low quality of life (8, 9). In addition, sarcopenia has reportedly been associated with an increased risk of mortality in the general population (10–13). On the other hand, few studies have investigated the association between sarcopenia and mortality in people with T2D (14, 15). Previous studies have evaluated the effect of low muscle strength (14) or low muscle mass (15) on mortality but not the effect of sarcopenia on mortality. Thus, the purpose of this prospective cohort study was to investigate the effect of sarcopenia, including both muscle mass loss and low muscle strength, on mortality in people with T2D.

## Materials and Methods

### Study Design, Setting, and Participants

This prospective cohort study was a part of the KAMOGAWA-DM cohort study, which has been conducted since 2014 (16). This cohort includes outpatients visiting the Department of Endocrinology and Metabolism, Kyoto Prefectural University of Medicine Hospital (Kyoto, Japan) and the Department of Diabetology, Kameoka Municipal Hospital (Kameoka, Japan). This study was approved by the local research ethics committee (No. RBMR-E-466-6) and was carried out in accordance with the ethical standards laid down in the 1964 Declaration of Helsinki and its later amendments. After obtaining written informed consent, medical data were anonymously gathered and compiled into a database. In this study, the data of all people with T2D were extracted from the database (17). Exclusion criteria were as follows: 1) no data on handgrip strength; 2) no data on body composition; 3) no data on potential covariates, such as medication, lifestyles, and metabolic parameters, including glycosylated hemoglobin A1c (HbA1c), creatinine, triglycerides, and blood pressure; 4) inaccurate data; and 5) people aged under 60 years (18).

### Data on Lifestyle Characteristics, Past Medical Histories, Medications, and Metabolic Parameters

On analyzing body composition, the following data were also obtained:

Lifestyle characteristics, such as exercise, smoking, and alcohol, were evaluated. “Habit of exercise” was defined as performing any kind of physical activity at least once a week, “habit of smoking” as currently smoking cigarettes or another tobacco product, and “habit of alcohol” as daily alcohol consumption.

Past medical histories regarding CVD, including angina, coronary heart disease, heart failure, prior acute myocardial infarction, stroke (ischemic or hemorrhagic), and cancers, were obtained from electronic medical records.

Data on diabetes and hypertension medications as well as on usage of steroids were obtained from electronic medical records. Duration of diabetes at baseline was determined based on any one of the following: declaration from people, the date of the first abnormal laboratory report on diabetes, or the date of the first diabetes-related treatment, whichever preceded.

Laboratory data, including creatinine, HbA1c, and triglycerides, were obtained from venous blood samples following overnight fasting. HbA1c was measured using high-performance liquid chromatography and was expressed in National Glycohemoglobin Standardization Program units. The estimated glomerular filtration rate (eGFR; mL/min/1.73 m^2^) was calculated using the Japanese Society of Nephrology equation (eGFR = 194 × serum creatinine^−1.094　^× age^−0.287^ × [0.739 for women]) (19).

Blood pressure measurement was performed automatically using an automatic blood pressure measurement device (HEM-906; OMRON, Kyoto, Japan) in a quiet space after 5 min of rest. The handgrip strength of each hand was measured using a handgrip dynamometer (Smedley, Takei Scientific Instruments Co., Ltd., Niigata, Japan), and the maximum value was used for analysis (20).

Body composition was evaluated using a multifrequency impedance body composition analyzer, InBody 720 (InBody Japan, Tokyo, Japan), which has been reported to have good correlation with dual-energy X-ray absorptiometry (21). Body weight (BW, kg), appendicular muscle mass (kg), and body fat mass (kg) were obtained. Body mass index (BMI, kg/m^2^) and skeletal muscle mass index (SMI, kg/m^2^) were calculated as follows: BW (kg) ÷ height squared (m^2^) and appendicular muscle mass (kg) ÷ height squared (m^2^), respectively.

### Definitions of Sarcopenia

Sarcopenia was defined based on the Asian Working Group for Sarcopenia guidelines, using handgrip strength and SMI (2). The cutoff values for handgrip strength indicating low muscle strength were <28 kg for men and <18 kg for women, and those indicating a low SMI were <7.0 kg/m^2^ for men and <5.7 kg/m^2^ for women. People who had both low muscle strength and low SMI were diagnosed with sarcopenia (2).

### Outcome of This Study and Follow-Up

The outcome of this study was the occurrence of all-cause mortality. Death events were obtained from medical records. The follow-up duration (month) was calculated from the date of examination at baseline to the time of death, date of last follow-up (for people who ceased hospital visits), date of transfer to other hospitals, or otherwise, up to January 2021.

### Statistical Analysis

Statistical analyses were performed using EZR (Saitama Medical Center, Jichi Medical University, Saitama, Japan) (22), which is a graphical user interface for R (The R Foundation for Statistical Computing, Vienna, Austria).

Mean (standard deviation) or frequencies of potential confounding variables were expressed. Participants were divided into two groups according to the presence or absence of sarcopenia. The differences in continuous and categorical values were evaluated using the Student’s t-test and chi-square or Fisher’s exact test, respectively. Correlations were analyzed using the Pearson’s correlation coefficient.

Kaplan–Meier analysis was performed to obtain a graphical representation of time against incident all-cause mortality, and a log-rank test was performed to assess the difference between participants with and without sarcopenia.

Due to the low incidence of all-cause mortality for statistical analysis, we used propensity scores to preserve statistical power by reducing the covariates into a single variable. To assess the propensity score, the dependent variable was the presence of sarcopenia. Propensity scores were evaluated using multivariable logistic regression models that included the following parameters: age, sex, duration of diabetes, history of heart disease, history of cancer, smoking, exercise, alcohol consumption, sodium-glucose cotransporter-2 (SGLT2) inhibitor, glucagon-like peptide-1 (GLP-1) receptor agonist, insulin, corticosteroid, hypertension, BMI, HbA1c, triglycerides, and creatinine. The C-statistic for the propensity score model was 0.89, which displays acceptable discrimination. Thereafter, the hazard ratio (HR) for the risk of incident all-cause mortality in the presence of sarcopenia was calculated using a Cox regression model.

Subsequently, we investigated the effect of low SMI or low muscle strength on incident all-cause mortality using Kaplan–Meier analyses and the Cox regression model.

## Results

**Figure 1:**
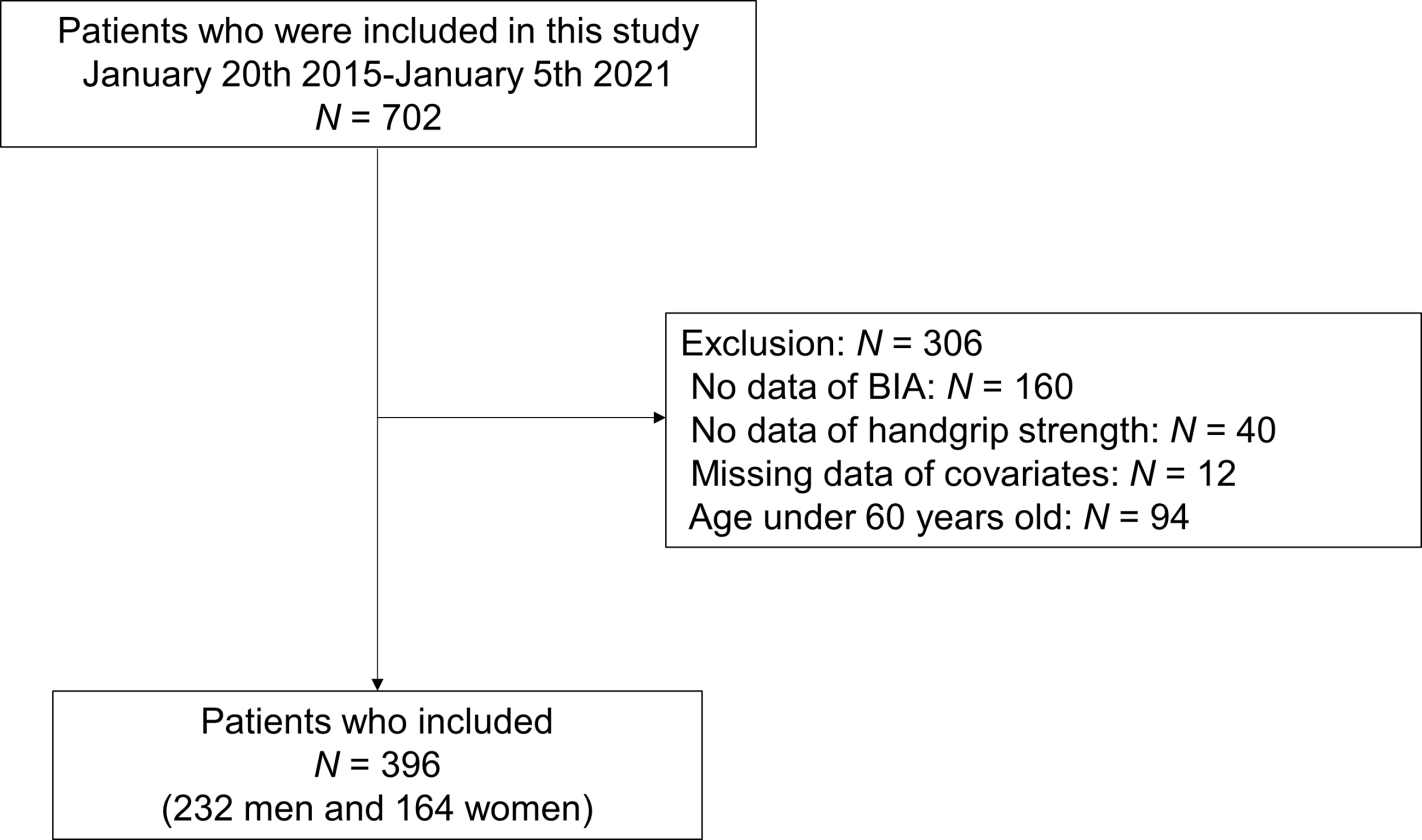
Study flow diagram for the registration of participants. BIA, bioimpedance analysis.

In this study, 702 people with T2D were recruited from the database, and 306 were excluded (**Figure 1**). Thus, 396 people were included in this study.

Among the 396 participants (232 men and 164 women), the average age and duration of diabetes were 71.3 (6.3) years and 16.3 (11.3) years, respectively (**Table 1**). The proportions of low muscle strength, low SMI, and sarcopenia were 26.8%, 28.3%, and 14.6%, respectively. During the average 40.5 (16.5) months of follow-up, 13 people died (cancers, 7; infections, 3; CVD, 2; and unknown, 1).

**Table 1:** Clinical characteristics of study participants with and without sarcopenia.

	All N = 396	Sarcopenia (-) N = 338	Sarcopenia (+) N = 58	*p*
Sex (men/women)	232/164	195/143	37/21	0.476
Age (years)	71.3 (6.3)	70.3 (5.8)	77.3 (5.7)	<0.001
Duration of diabetes (years)	16.3 (11.3)	15.4 (11.0)	21.4 (11.9)	<0.001
Family history of diabetes (-/+)	238/158	198/140	40.18	0.178
Height (cm)	160.6 (8.7)	161.1 (8.7)	157.7 (8.3)	0.005
Body weight (kg)	61.1 (10.8)	62.6 (10.5)	52.2 (8.4)	<0.001
Body mass index (kg/m^2^)	23.6 (3.7)	24.1 (3.6)	20.9 (2.6)	<0.001
Systolic blood pressure (mmHg)	133.5 (18.3)	134.1 (18.3)	130.4 (18.3)	0.164
Diastolic blood pressure (mmHg)	75.4 (11.8)	76.6 (11.2)	68.5 (13.1)	<0.001
Antihypertensive drugs (-/+)	166/230	144/194	22/36	0.673
Presence of hypertension (-/+)	121/275	104/234	17/41	0.945
SGLT2 inhibitor (-/+)	339/57	286/52	53/5	0.249
GLP-1 receptor agonist (-/+)	342/54	292/46	50.8	1.000
Insulin (-/+)	295/101	254/84	41/17	0.578
Corticosteroids (-/+)	382/14	330/8	52/6	0.009
History of heart disease (-/+)	320/76	283/55	37/21	<0.001
History of cancer (-/+)	331/65	283/55	48/10	1.000
Habit of smoking (-/+)	337/59	286/52	51/7	0.649
Habit of exercise (-/+)	198/198	175/163	23/35	0.118
Habit of drinking alcohol (-/+)	277/119	236/102	41/17	1.000
Hemoglobin A1c (%)	7.3 (1.0)	7.2 (1.1)	7.4 (1.0)	0.294
Hemoglobin A1c (mmol/mol)	55.9 (11.5)	55.6 (11.5)	57.3 (10.9)	0.294
Plasma glucose (mmol/L)	147.7 (47.3)	8.1 (2.7)	8.5 (2.5)	0.310
Creatinine (µmol/L)	76.3 (33.7)	76.3 (33.9)	76.6 (33.0)	0.943
eGFR (mL/min/1.73 m^2^)	66.3 (18.3)	66.3 (18.1)	66.3 (19.9)	0.986
Triglycerides (mmol/L)	1.4 (0.9)	1.5 (0.9)	1.3 (0.6)	0.143
HDL cholesterol (mmol/L)	1.6 (0.5)	1.6 (0.4)	1.5 (0.5)	0.701
Handgrip strength (kg)	27.1 (8.7)	28.4 (8.5)	19.7 (6.1)	<0.001
Low muscle strength (-/+)	290/106	290/48	0/58	<0.001
Appendicular muscle mass (kg)	17.9 (3.9)	18.4 (3.8)	14.9 (3.2)	<0.001
Skeletal muscle mass (kg/m^2^)	6.9 (1.0)	7.0 (0.9)	5.9 (0.8)	<0.001
Low skeletal muscle mass (-/+)	284/112	284/54	0/58	<0.001
Incident mortality (-/+)	383/13	332/6	51/7	<0.001
1000 patient years of follow-up	1.34	1.17	0.17	<0.001
Incident mortality/ 1000 patient years of follow-up	9.72	5.14	41.3	<0.001

Data was expressed as mean (standard deviation) or number. The difference between group was evaluated by Student's t-test, chi-square test or fisher’s exact test. eGFR, estimated glomerular filtration rate; and HDL, high-density lipoprotein.

SMI was negatively correlated with age (*r* = -0.207, *p <*0.001) and duration of diabetes (*r* = -0.106, *p* = 0.036). Handgrip strength was negatively correlated with age (*r* = -0.286, *p <*0.001) and duration of diabetes (*r* = -0.141, *p* = 0.005). BMI was positively associated with SMI (*r* = 0.512, *p <*0.001) and handgrip strength (*r* = 0.103, *p* = 0.040). On the other hand, hemoglobin A1c was not correlated with both SMI (*r* = -0.077, *p* = 0.127) and handgrip strength (*r* = 0.002, *p* = 0.963) (**Table 2**).

**Table 2:** The correlation between the variables of diabetes and skeletal muscle mass or handgrip strength.

	Skeletal muscle mass (kg/m^2^)		Handgrip strength (kg)	
	*r*	*p*	*r*	*p*
Age (years)	-0.207	<0.001	-0.286	<0.001
Duration of diabetes (years)	-0.106	0.036	-0.141	0.005
Body mass index (kg/m2)	0.512	<0.001	0.103	0.040
Hemoglobin A1c (%)	-0.077	0.127	0.002	0.963
Hemoglobin A1c (mmol/mol)	-0.077	0.127	0.002	0.963
Plasma glucose (mmol/L)	-0.087	0.086	-0.037	0.461

Correlations were analyzed using the Pearson’s correlation coefficient.

**Figure 2:**
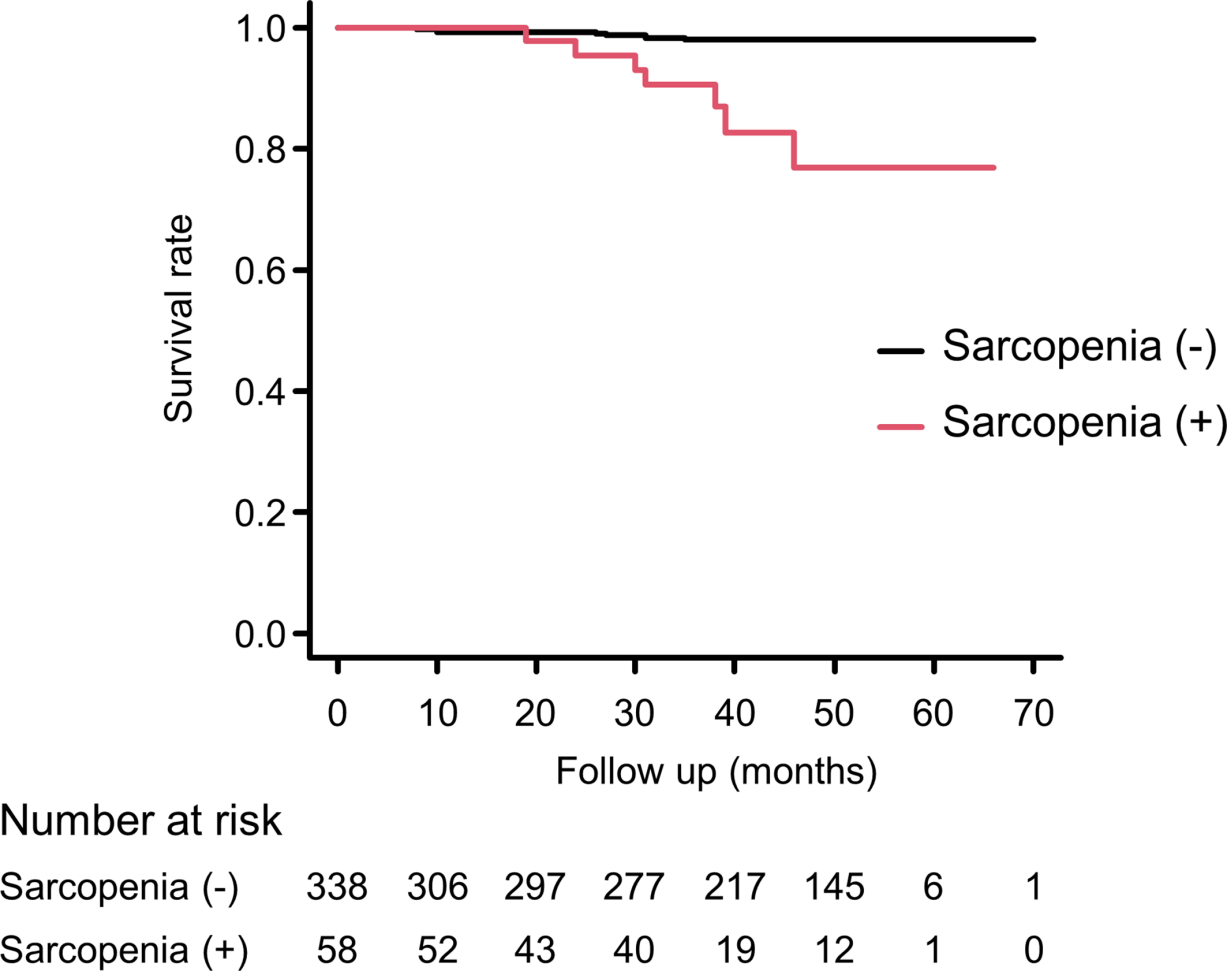
Kaplan-Meier survival curve according to the presence or absence of sarcopenia. According to the log-rank test, *p < *0.001.

**Figure 2** shows results of the Kaplan–Meier curve for cumulative survival rate according to the presence or absence of sarcopenia. The presence of sarcopenia was associated with higher mortality risk (log-rank test, *p <*0.001). Sarcopenia was also associated with all-cause mortality after adjusting for covariates (HR, 6.12; 95% confidence interval [CI], 1.52–24.7; *p* = 0.011) (**Table 3**).

**Table 3:** Hazard ratio of the presence or absence of sarcopenia, low muscle strength or low skeletal muscle mass for the all-cause mortality.

		Model 1		Model 2	
		HR (95 %CI)	*p*	HR (95 %CI)	*p*
Sarcopenia	(-)	Ref	–	Ref	–
	(+)	8.86 (2.96-26.5)	<0.001	6.12 (1.52-24.7)	0.011
Low muscle strength	(-)	Ref	–	Ref	–
	(+)	11.5 (3.16-41.9)	<0.001	8.76 (2.16-35.5)	0.002
Low skeletal muscle mass	(-)	Ref	–	Ref	–
	(+)	6.38 (1.96-20.7)	0.002	4.14 (1.08-15.9)	0.039

Since the cases of mortality is not enough. Propensity score was used for covariates. Propensity score was evaluated by multivariable logistic regression models that include the age, sex, duration of diabetes, history of heart disease, history of cancer, smoking, exercise, drinking alcohol, SGLT2 inhibitor, GLP-1 receptor agonist, insulin, corticosteroid, hypertension, body mass index, hemoglobin A1c, triglycerides, and creatinine. The sarcopenia for propensity score model was 0.89. Model 1 was unadjusted. Model 2 was adjusted for the propensity score.

**Figure 3:**
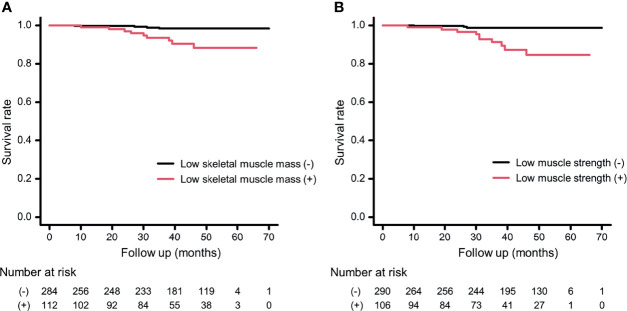
Kaplan-Meier survival curve according to the presence or absence of low skeletal muscle mass or low muscle strength. **(A)** The presence or absence of low skeletal muscle mass. **(B)** The presence or absence of low muscle strength. According to the log-rank test, both *p < *0.001.

**Figure 3** shows the results of the Kaplan–Meier survival curve for cumulative survival rate according to the presence or absence of low muscle strength or low SMI. The presence of sarcopenia, low muscle strength, and low SMI were associated with a higher mortality risk (both log-rank test, *p <*0.001). In addition, both low muscle strength and low SMI were associated with all-cause mortality after adjusting for covariates (low muscle strength: HR, 8.76; 95% CI, 2.16–35.5), *p* = 0.002; and low SMI: HR, 4.14; 95% CI, 1.08–15.9, *p* = 0.039) (**Table 2**).

## Discussion

The main finding of this study was that sarcopenia is associated with incident all-cause mortality in older outpatients with T2D after adjusting for covariates. In addition, sub-analysis revealed that both low handgrip strength and low SMI were also associated with incident all-cause mortality in older outpatients with T2D, a finding similar to that of previous studies (14, 15). The association between low muscle strength (14) or low muscle mass (15) and mortality in old people with T2D has been reported; however, there are no studies reporting an association between sarcopenia, defined by both low muscle strength and low muscle mass, and mortality in old people with T2D.

Previous meta-analyses have revealed that sarcopenia is a risk factor for morality not only in community-dwelling older individuals (11, 23) but also in older nursing-home residents (24). Moreover, recent meta-analyses have revealed that sarcopenia is a mortality risk in people with colorectal cancer (25). In addition to these meta-analyses, recent studies have confirmed that sarcopenia is a risk factor for morality in people on hemodialysis (5) and in older hospitalized people (12). However, there are no reports investigating the association between sarcopenia, defined by both low muscle strength and low muscle mass, and mortality in people with T2D. These results were consistent with those from our study.

The possible mechanisms behind the association between sarcopenia and mortality risk are as follows. Sarcopenia has reportedly been linked to decreasing protein synthesis and increasing protein degradation, through increased oxidative stress (26), decreasing antioxidant defenses (26), and inflammations (27), leading to mortality (28). These factors are also associated with atherosclerosis. In fact, sarcopenia, especially low muscle strength, has been associated with blood pressure variability (29) and risk of cardiovascular events (4–6). In addition, sarcopenia has reportedly been associated with a high risk of malnutrition (30, 31), which aggravates sarcopenia, resulting in an increased mortality rate. Furthermore, although we adjusted for cancer as a covariate in this study, cachexia, characterized by systemic inflammation and abnormal metabolism with loss of muscle mass as well as reduced food intake, is often accompanied by sarcopenia in people with cancer (18). Thus, sarcopenia in older people with cancer has been shown to be predictive of a negative clinical outcome, due not only to the loss of tissue mass but also the loss of cell integrity (32, 33).

The treatment and prevention of sarcopenia is an important issue in T2D because the prevalence of sarcopenia has been reported to be higher in T2D than in the general population (3). In fact, among our study participants, the prevalence of sarcopenia was 15.1%, which was almost similar to that of previous studies (34, 35). Exercise training as well as adequate protein and calorie intakes are necessary to maintain and increase muscle mass (36–40). Furthermore, handgrip exercises decrease vascular resistance by a cholinergic mechanism (41), although it is possible that such a physiological effect may be attenuated in people with T2D because of autonomic nervous dysfunction. In addition, good glycemic control (35) and proper usage of medications, including insulin (35), GLP-1 receptor agonist (42, 43), and SGLT2 inhibitor (44, 45), have been reported to improve sarcopenia.

The limitations of this study should not go unmentioned. First, we used the multifrequency impedance body composition analyzer for body composition analysis, although dual-energy X-ray absorptiometry is the gold standard method for evaluating skeletal muscle mass. A previous study reported that bioimpedance analysis overestimated fat mass and underestimated lean soft tissue mass when validated against dual-energy X-ray absorptiometry (21). Second, the incidence of all-cause mortality was relatively small. Thus, we could not perform a detailed analysis of the cause of death. The number of outcomes was relatively small, further follow-up is imperative. Third, vitamin D status, including serum vitamin D level and vitamin D intake, did not assess in this study, although vitamin D status was reported to be associated with muscle mass loss (46) and muscle mass strength (47). Fourth, this study included only people with T2D; generalization to the other groups is hence uncertain. Finally, this study only included Japanese individuals. Therefore, it is possible that the results of this study may not be generalized to individuals from other backgrounds.

In conclusion, sarcopenia is associated with incident all-cause mortality in older outpatients with T2D. The present findings contribute to our understanding of the importance of sarcopenia in preventing future mortality in older people with T2D.

## Data Availability Statement

The datasets used and/or analyzed during the current study are available from the corresponding author on reasonable request.

## Ethics Statement

The studies involving human participants were reviewed and approved by Kyoto prefectural University of Medicine. The patients/participants provided their written informed consent to participate in this study.

## Author Contributions

FT obtained, analyzed, and interpreted data and wrote the manuscript. YH planned and designed the work, obtained, analyzed, and interpreted data, and wrote the manuscript. AK, RS, and TO obtained and interpreted data and contributed to the discussion. NK, HO, NN, SM, and TS obtained data and contributed to the discussion. EU and MH planned the work, obtained data, and contributed to the discussion. MA and MY obtained data and contributed to the discussion. MF planned and designed the work, obtained and interpreted data, and contributed to the discussion. All authors have checked the final version, and agree to be responsible for the work to ensure that any questions related to the accuracy or completeness of any of the work are appropriately investigated and resolved.

## Conflict of Interest

YH reports personal fees from Novo Nordisk Pharma Ltd., Daiichi Sankyo Co. Ltd., Sanofi K.K., Mitsubishi Tanabe Pharma Corp., Ono Pharma Co., Ltd., Takeda Pharma Co., Ltd., and Sumitomo Dainippon Pharma Co. Ltd. EU received personal fees from MSD K.K., Mitsubishi Tanabe Pharma Corp., AstraZeneca plc, Daiichi Sankyo Co. Ltd., Novo Nordisk Pharma Ltd., Taisho Toyama Pharma Co., Ltd., Kowa Pharma Co. Ltd., Takeda Pharma Co., Ltd., Kyowa Kirin Co. Ltd., Sumitomo Dainippon Pharma Co. Ltd., Astellas Pharma Inc., and Nippon Boehringer Ingelheim Co. Ltd. outside the submitted work and received grant support from the Astellas Foundation for Research on Metabolic Disorders and the Japanese Study Group for Physiology and Management of Blood Pressure, donated fund Laboratory of Diabetes therapeutics is an endowment department, supported with an unrestricted grant from Ono Pharma. Co., Ltd. MH received grants from Daiichi Sankyo Co. Ltd., Astellas Pharma Inc., Mitsubishi Tanabe Pharma Corp., Novo Nordisk Pharma Ltd., Nippon Boehringer Ingelheim Co. Ltd., Sanofi K.K., Takeda Pharma Co. Ltd, Sumitomo Dainippon Pharma Co. Ltd., Asahi Kasei Pharma, Kyowa Kirin Co. Ltd., and Eli Lilly Japan K.K., outside the submitted work. MA received personal fees from Takeda Pharmaceutical Co., Ltd., Abbott Japan Co., Ltd., Sumitomo Dainippon Pharma Co., Ltd., Kowa Pharmaceutical Co., Ltd., Novo Nordisk Pharma Ltd., Ono Pharmaceutical Co., Ltd., AstraZeneca K.K., and Chugai Pharmaceutical Co., Ltd., outside the submitted work. MY received personal fees from Ono Phama Co., Ltd., Kowa Pharma Co. Ltd., AstraZeneca plc., Sumitomo Dainippon Pharma Co. Ltd., Kyowa Kirin Co. Ltd., MSD K.K., Takeda Pharma Co. Ltd, Kowa Pharma Co. Ltd., and Daiichi Sankyo Co. Ltd. outside the submitted work. MF received grants from Taisho Pharma Co., Ltd., Mitsubishi Tanabe Pharma Corp, Novo Nordisk Pharma Ltd., Ono Pharma Co. Ltd., Kowa Pharma Co. Ltd., Sanofi K.K., Nippon Boehringer Ingelheim Co. Ltd., Daiichi Sankyo Co. Ltd., Kissei Phama Co. Ltd., MSD K.K., Kyowa Kirin Co., Ltd., Sumitomo Dainippon Pharma Co., Ltd., Eli Lilly Japan K.K., Tejin Pharma Ltd., Takeda Pharma Co. Ltd., Nippon Chemiphar Co., Ltd., Astellas Pharma Inc., Abbott Japan Co. Ltd., Sanwa Kagagu Kenkyusho CO., LtD., Johnson & Johnson k.k. Medical Co., and Terumo Corp., and received honoraria from AstraZeneca K.K., Taisho Pharma Co., Ltd., Ono Pharma Co. Ltd., Novo Nordisk Pharma Ltd., Sanofi K.K., Teijin Pharma Ltd., Takeda Pharma Co. Ltd., Astellas Pharma Inc., MSD K.K., Mitsubishi Tanabe Pharma Corp., Eli Lilly Japan K.K., Kissei Pharma Co., Ltd., Sumitomo Dainippon Pharma Co. Ltd., Daiichi Sankyo Co. Ltd., Mochida Pharma Co. Ltd., Kowa Pharma Co. Ltd., Arkray Inc., Abbott Japan Co. Ltd., Sanwa Kagaku Kenkyusho Co. Ltd., Kyowa Kirin Co. Ltd., Nippon Boehringer Ingelheim Co., Ltd., Medtronic Japan Co. Ltd., Bayer Yakuhin, Ltd., and Nipro Corp. outside the submitted work.

The remaining authors declare that the research was conducted in the absence of any commercial or financial relationships that could be construed as a potential conflict of interest.

## Publisher’s Note

All claims expressed in this article are solely those of the authors and do not necessarily represent those of their affiliated organizations, or those of the publisher, the editors and the reviewers. Any product that may be evaluated in this article, or claim that may be made by its manufacturer, is not guaranteed or endorsed by the publisher.
